# Potential and Comparative Tablet Disintegrant Properties of Pectin Obtained from Five Okra Genotypes in Ghana

**DOI:** 10.1155/2021/2902335

**Published:** 2021-06-19

**Authors:** Frederick William Akuffo Owusu, Mariam El Boakye-Gyasi, Jacob Kwaku Agbenorhevi, Marcel Tunkumgnen Bayor, Kwabena Ofori-Kwakye

**Affiliations:** ^1^Department of Pharmaceutics, Faculty of Pharmacy and Pharmaceutical Sciences, Kwame Nkrumah University of Science and Technology, Kumasi, Ghana; ^2^Department of Food Science and Technology, College of Science, Kwame Nkrumah University of Science and Technology, Kumasi, Ghana

## Abstract

Okra pectin has been studied as a potential excipient in tablet formulations for pharmaceutical industries. Okra is widely grown and available in Ghana and other parts of the world. The prospective use of pectin from okra genotypes grown in Ghana as tablet disintegrants has not been reported. This study aims to determine the potential and comparative disintegrating properties of pectin from five okra genotypes (*Abelmoschus esculentus* L.) in Ghana using uncoated immediate release paracetamol tablet formulations. The yield of the pectin from the various genotypes ranged between 6.12 and 18.84% w/w_._ The extracted pectins had pH ranging from slightly acidic to almost neutral (6.39–6.92). Pectin from the various genotypes exhibited good swelling indexes (˃200%), varying solubility in different solvents, and low moisture content (˂20%). Elemental analysis of the extracted pectin from the various genotypes revealed very low levels of toxic metals and micronutrients. Pectin from the various genotypes was evaluated as disintegrants within concentrations of 5–10% w/w (F1–F18). Their disintegrating properties were compared to that of maize starch BP. All the formulated batches of uncoated immediate release paracetamol tablets (F1–F18) passed the following: uniformity of weight test, uniformity of dimensions, hardness, friability (˂1%), and drug content (95–105%). Significant differences (*p* ≤ 0.05) were observed between the hardness of the maize starch tablets and tablets formulated from pectin of the various genotypes. Pectin from all genotypes other than PC5 exhibited good disintegrating properties (*D*_*T*_ ˂ 15 min) and subsequently passed the dissolution profile test (≥70% release in 45 minutes). Tablets formulated with PC5 as disintegrants at all concentrations (5% w/w (F5), 7.5% w/w (F11), and 10% w/w (F17)) failed the disintegration and dissolution tests. Ultimately, pectins extracted from PC1, PC2, PC3, and PC4 can be commercially exploited as disintegrants in immediate release tablets.

## 1. Introduction

Disintegrants are pharmaceutical excipients that are added to immediate release tablet formulations to facilitate their breakup into smaller fragments when in contact with the aqueous medium of the gastrointestinal tract. This aids in faster release and subsequent absorption of the active pharmaceutical ingredient (API) [1, 2]. Immediate release tablets do not serve their purpose unless they spontaneously and in a predictable manner release their API for absorption in the gastrointestinal fluid. Thus, disintegrants are undoubtedly one of the indispensable excipients needed in achieving the ideal property of immediate release tablets.

Disintegrants can be added to a granule or powder mix either intragranularly, extragranularly, or as a combination of both [3]. Even though several mechanisms of disintegration, such as swelling, particle-particle repulsive force, wicking, and deformation, have been studied, it has also been reported that the type of disintegrant, concentration, and mode of incorporation of a disintegrant affects its disintegrating properties and tablet characteristics such as hardness and friability [4]. Disintegrants can be obtained from natural and synthetic sources. The use of natural polymeric excipients in the pharmaceutical sector is increasing on a daily basis over semisynthetic or synthetic excipients due to their low toxicity, availability, cost-effectiveness, and nonirritant nature [5, 6].

Conventional sources of disintegrants are known to pose several disadvantages such as tablet softening [7, 8]. Relentless efforts have therefore been channeled into obtaining disintegrants from natural sources such as gums and mucilage as an alternative to conventional sources. This has resulted in the development of novel sources of disintegrants such as khaya gum, *Moringa oleifera* gum, *Azadirachta indica* gum, and *Sterculia urens* gum [9]. However, most of these novel sources of disintegrants are trees and shrubs which have long maturity periods and require large land space for cultivation. There is therefore the need to search for other potential natural sources of disintegrants which will ameliorate the challenges of sources of gums and mucilage as disintegrants. A natural polysaccharide which can be an alternative source of disintegrant due to its water absorbing and swelling properties but has been given less attention is pectin.

Pectin is a family of complex polysaccharides that are found in the primary cell wall and middle lamella of dicotyledons. Unlike the major sources of gums and mucilage, pectin can be sourced from vegetables and lower plants which have shorter maturity periods and require smaller land space for cultivation such as okra. Pectin can therefore be utilized as an alternative source of pharmaceutical excipients at a cheaper cost. The food and beverage industry is already harnessing the properties of pectin as a thickening agent, a gelling agent, and a colloidal stabilizer in their products [5, 10]. Pectin obtained from the citrus family has been investigated for its binding properties in tablets [11, 12]. However, currently, there is little or no available literature on the disintegrating properties of pectin. It has been reported that sourcing pectins from plants with short maturity and investigating their disintegrating and other tablet formulation properties will diversify their use in the pharmaceutical industry and attract huge revenues for pharmaceutical industries involved in this research [13, 14].

Okra is cultivated in Ghana and many parts of the world mainly for its nutritional value. Its potential for supplying high yields of pectin for pharmaceutical use is a promising area for pharmaceutical scientists to explore [15]. Even though pectin from okra has been investigated and shown to have good drug release modifying properties, there is little information on the potential binding and disintegrating properties of okra pectin [16–19]. Several genotypes of okra are available in Ghana and are widely cultivated in almost every region with variations in duration of cultivation, time of maturation, pod size, and shape [20]. The aim of this current study is to investigate the potential and comparative disintegrant properties of pectin from five (5) okra genotypes cultivated in Ghana using immediate release paracetamol tablet formulations. Investigating the disintegrating properties of okra pectin from different genotypes will provide the needed literature on the potential of pectins to be used as disintegrants and the effect of genotypes on the quality of pectin as a disintegrant. Ultimately, the potential for commercialization of okra being cultivated in Ghana as a pharmaceutical excipient (disintegrants) can be determined from this study.

## 2. Materials and Methods

### 2.1. Materials

The pods of five okra genotypes (*Abelmoschus esculentus* L), namely, Penkrumah, Agbagoma, Asha, Sengavi, and Balabi, (PC1, PC2, PC3, PC4, and PC5) were obtained from the Ghanaian market and authenticated at the Department of Horticulture, KNUST, Ghana. Maize starch BP (UK Chemicals) was used as the reference disintegrant in formulations coded F6, F12, and F18. Tragacanth BP (Sigma-Aldrich), paracetamol BP (Xi'an Henrikang Biotech Co., China), lactose, talc, disodium hydrogen phosphate, and sodium dihydrogen phosphate (VWR Chemicals, UK) were used. All other reagents and chemicals used were of analytical grade.

### 2.2. Methodology

#### 2.2.1. Extraction and Determination of Pectin Yield

The okra pods were cut open and the seeds removed. The separated okra pods were sun-dried, milled to powder, and then stored in zip-lock bags pending extraction. Okra pectin was isolated and extracted at pH 6.0 according to the previous extraction protocol [20, 21]. The pectin yield was calculated based on the amount of dry okra powder used for the extraction process and the amount of freeze-dried pectin obtained after extraction. The percentage yield (w/w) was calculated on a dry weight basis [20].

#### 2.2.2. Physicochemical Properties of Extracted Pectin

The pH, moisture content, swelling index, solubility, and elemental contents of pectin from the various genotypes were determined using official methods [12, 22–25].

#### 2.2.3. Drug-Excipient Compatibility Studies

The compatibility between paracetamol and pectin from the various genotypes was determined using a Fourier transform infrared spectrometer (Bruker alpha II). Paracetamol spectra, individual pectin, and physical mixtures of paracetamol and pectin were recorded by scanning in the wavelength region of 4000–400 cm^−1^ using the FTIR spectrometer. The spectra of the three samples were subsequently superimposed to assess if the main absorption bands present in the drug and pectin are still evident in the physical mixtures.

#### 2.2.4. Preparation of Tablets

Eighteen different batches of granules were prepared by the wet granulation method. The composition of formulated tablets is shown in Table 1 (pectin from various genotypes were used as follows: disintegrants at different concentrations: ∼5% w/w (F1–F5), ∼7.5% w/w (F7–F11), ∼10% w/w (F13–F17). A blend of all ingredients except the talc (lubricant and glidant) was mixed in a porcelain mortar. Half of the disintegrant was added intragranularly and the rest extragranularly. Kneading was done using a mucilage of water and the binder until a damp mass was formed. The damp mass was screened through a 2360 *µ*m mesh and the wet granules were dried at 60°C for 1 hour in a hot air oven. The dried granules were screened through a 1190 *µ*m mesh. The granules were kept in airtight containers and stored in a desiccator pending further analysis and subsequent compression into tablets. The angle of repose was determined using the fixed height method. The bulk and tapped densities were used for the determination of compressibility index as well as Hausner's ratio [3]. Talc was incorporated by hand mixing for 5 minutes just before compression. The granules were compressed into tablets using a single punch tableting machine (Saimach press 11/37, India).

#### 2.2.5. Evaluation of Tablet Properties

*(1) Physicomechanical Properties*. The physicomechanical properties of formulated tablets such as weight, dimensions, friability, crushing strength, and disintegration times were assessed using Pharmacopoeia methods [22, 26, 27]

*(2) Assay*. The content of paracetamol in each of the eighteen batches was determined using a reverse-phase chromatographic technique developed and validated in [28] with some modifications. Twenty paracetamol tablets were randomly sampled and weighed accurately and the average weight was recorded. The tablets were crushed to a fine powder and a quantity of the powder equivalent to 0.15 g of paracetamol was accurately weighed. The powder mixture was dissolved in a mobile phase (methanol 65%: 0.1% TFA in water 35%) with the aid of sonication and then made up to the 100 ml mark with the mobile phase. The solution was filtered through Whatman filter paper (No. 5) into another 100 ml volumetric flask. From the above filtrate, 1 ml was taken in a 10 ml volumetric flask and volume was made up to the mark with the mobile phase; the solution was then filtered using sintered glass filter and loaded in the injector of an Agilent HPLC (1260 with programmable absorbance detector and Agilent Zorbax SB-Phenyl 150 mm × 3.0 mm × 3.5 *μ*m column). The sample solution (1 *μ*l) was injected at a flow rate of 1 ml/min and the detection of eluent was carried out at 230 nm. The injection was repeated three times and the peak area of paracetamol was recorded. The average peak area was then used to calculate the amount of drug present using the average peak area of pure paracetamol with the same concentration as a standard. The experimental procedure was repeated for the other batches.

*(3) In Vitro Dissolution Test*. The Veego UDA-8D USP dissolution apparatus II (paddle apparatus) was used to conduct the dissolution test. Phosphate buffer (900 ml) with a pH of 5.8 was used for the dissolution test of the tablets. The temperature of the medium was strictly maintained at 37 ± 2°C throughout the experiment. One dosage unit from a batch was placed in each of the six vessels and the stirrers and timer switched on simultaneously. The speed of agitation used was 50 rpm. Ten (10) ml of the dissolution media was withdrawn and filtered at different time intervals of 5, 15, 30, 45, and 60 minutes. An equal volume (10 ml) of fresh medium having the same temperature was replaced at each time to maintain the sink condition in the vessel. Each sample withdrawn was filtered and 0.50 ml of each filtrate diluted to 50 ml with the phosphate buffer. The diluted solution was then assayed spectrophotometrically at 245 nm, using phosphate buffer (pH 5.8) in the reference cell. The amount of paracetamol released was then determined from the calibration curve (*y* = 806.29*x* − 0.0784, *R*^2^ = 0.9995. The experimental procedure was repeated for the other batches. A graph of percentage drug released against time was plotted to establish the dissolution profile of paracetamol from each batch [26, 29].

### 2.3. Statistical Analysis

The results are presented as the mean ± standard deviation. Data was analyzed using GraphPad Prism version 6.00 for Windows (GraphPad Software, San Diego California, USA). At 95% confidence interval, *p* ≤ 0.05 was considered significantly different. Dissolution profiles were compared using similarity (*f*2) and difference (*f*2) factors.

## 3. Results and Discussion

### 3.1. Extraction and Determination of Physicochemical Properties of the Okra Pectins

All five okra (*Abelmoschus esculentus* L.) genotypes after extraction produced pectin yields ranging from 6.12% to 18.84% w/w in the following order PC2 > PC5 > PC3 > PC4 > PC1. Pectin yield is known to be affected by the genotype used, the extraction method used, plant source, and maturation stage [20, 21]. Since the same extraction protocol was used, the differences in the pectin yield indicate structural variations in the pectin backbone of the various genotypes. Pectin from various genotypes of okra had slightly acidic to neutral pH (Table 2). These pH values will not cause irritation to the mucosal lining of the gastrointestinal tract. Similarly, these pH values do not alter the pH of the medium in which the active ingredient of the dosage form is dissolved and therefore do not affect the release profile of the active ingredient.

The intrinsic swelling properties of pectin from the various genotypes can be used to predict their potential mechanism of disintegration. The swelling capacity of pectin from the various genotypes was in the following order: PC2 > PC5 > PC4 > PC3 > PC1 (Table 2). The swelling property of okra pectin is attributed to the high concentrations of D-galacturonic acid polysaccharide backbone [30]. Variations in the genotypes resulted in differences in their swelling indexes. However, the swelling indexes of all the okra genotypes were higher than those reported of pectin from other sources [12, 25].

Variations in the chemical properties of pectin from the different genotypes resulted in differences in their solubility profile. This is similar to the results reported for the solubility profile of pectin obtained from mango and apple [31, 32]. The moisture content of powders and granules has an effect on their flow properties; high moisture content can cause granules to clamp together and agglomerate causing them to have poor flow. Microbial growth is also encouraged if moisture contents are high. All the genotypes had their moisture content being less than 20% and lesser than the moisture content reported for pectin from other sources [12].

**Figure 1:**
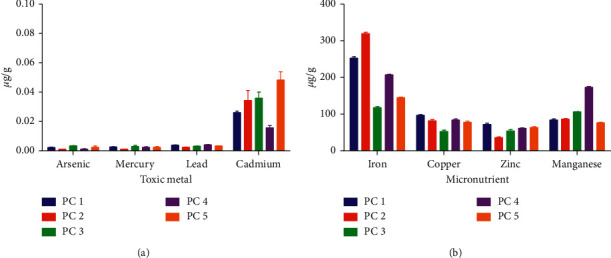
Elemental analysis on pectin from various genotypes; (a) toxic metals and (b) micronutrients.

Elemental analysis of pectin from different genotypes showed extremely low levels of toxic metals (Figure 1(a)). Micronutrient levels were also within acceptable limits (Figure 1(b)) [27]. This indicates the potential nontoxicity of okra pectin from the various genotypes and their suitability for use as pharmaceutical excipients.

**Figure 2:**
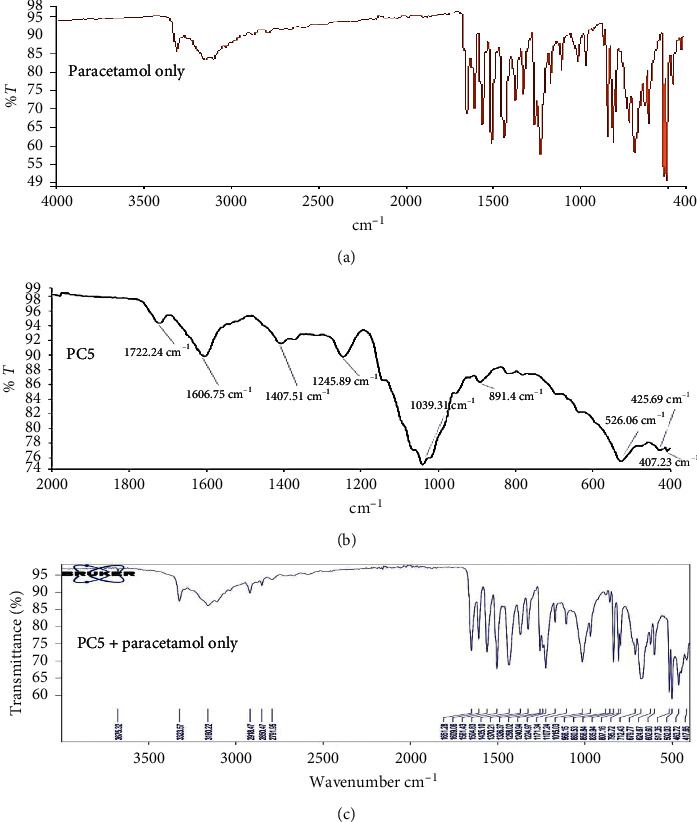
FTIR spectra of pure paracetamol (active), okra pectin (PC5), and the physical mixture of paracetamol and okra pectin (PC5 + active).

IR spectra of pectin from all the genotypes showed the following characteristic features: stretching within the range of 3100–3600 cm^−1^ (O-H stretching), bands within the range of 3000–2700 cm^−1^ (C-H stretching), and bands ranging from 1600 to 1670 cm^−1^ corresponding to COOH stretching. These bands were similar to those reported by Kpodo et al. and Alba et al. in the identification of okra pectin [20, 21]. A spectrum of pure paracetamol produces functional groups at 3322.03 cm^−1^, 3159.39 cm^−1^ (hydroxyl group, O-H stretching) and 1561.11 cm^−1^, 1504.98 cm^−1^ (amide II band). The physical mixtures contained all of the two constituents' main bands and showed no peak shifts, indicating the pectins' stability and compatibility with the active pharmaceutical ingredient (Figure 2).

### 3.2. Physicomechanical Properties of the Formulated Paracetamol Tablets

A summary of the physical and mechanical properties of the formulated paracetamol batches is shown in Tables 3 and 4 .

Based on the results shown (Table 3), the granules for compression generally exhibited good flow properties. The Hausner ratio value ranged from 1.01 to 1.18 suggesting a good and excellent flow of the granules. Carr's index ranged from 2.21% to 15.87% which suggests an excellent and good flow of the granules. The angle of repose obtained also ranged from 29.00° to 33.02°. This shows that the granules had a good flow [3]. Low moisture content of granules, relatively smooth surface of granules, spherical shapes, and adequate size distribution of granules are responsible for the good and excellent flow properties exhibited by the granules. The excellent and good flow property of granules indicates that a glidant concentration of ≤1% will be needed in the formulation of the tablets.

The total tablet weight produced was between 0.620 ± 0.001 and 0.639 ± 0.002 (Table 4). None of the tablet batches failed the uniformity of weight test (<2 tablets ± 5% mean weight, none ± 10% mean weight, *n* = 20) [25]. The consistent results may be attributed to the good flow properties of the prepared granules, the uniform compression force used in tablet compression, even feeding of granules into the die, and the regular movement of the lower punch to ensure a consistent weight distribution of the tablets [33].

Tablet thickness and diameter are very crucial factors in tablet packaging and aesthetic appeal: tablets which are too thick will affect the ease of blister and plastic packaging and invariably reduce patient compliance [34, 35]. All the formulated batches had their thickness and diameter within the accepted range of ±5%. This indicates consistency in dimensional characteristics of the formulated tablets in each batch and ultimately good compressional properties of okra pectin.

A tablet's resistance to abrasion, capping, and splitting during transportation and packaging depends on its friability and hardness. Immediate release tablets possessing friable properties may laminate or cap into halves during packaging whiles overly compact tablets may not disintegrate within time to meet the dissolution requirements [22, 26]. A minimum crushing force of 3 kg/f is required of tablets formulated as immediate release [36]. Thus, all the formulated batches will have good resistance to fracture since their crushing strengths were above the minimum value (Table 4) and confirms the suitability of okra pectins as pharmaceutical excipients in the compression of tablets.

**Figure 3:**
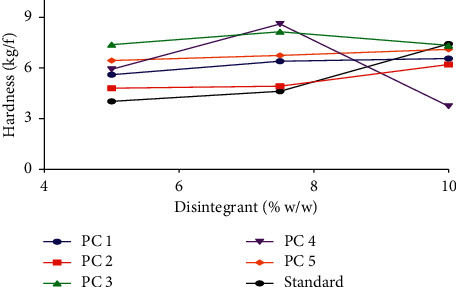
Effect of pectin concentrations as disintegrants on tablet hardness.

**Figure 4:**
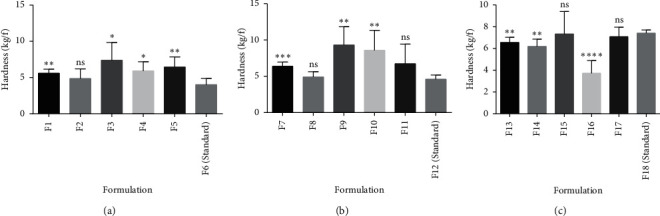
Comparative analysis on the crushing strength of pectin from various genotypes as disintegrants ((a) 5% w/w, (b) 7.5% w/w, and (c) 10% w/w) and a standard disintegrant using *t*-test. Values are mean ± SD (*n* = 6). ^*∗*^*p* ≤ 0.05, ^*∗∗*^*p* ≤ 0.01, ^*∗∗∗*^*p* ≤ 0.001, ^*∗∗∗∗*^*p* ≤ 0.0001, and *p* > 0.05 not significant (ns).

Excipients such as disintegrants have been reported to have an effect on the crushing strength of tablets [37–39]. Depending on the physicochemical properties of the disintegrant, increasing the disintegrant concentration may increase the hardness of the tablet and decrease the hardness of the tablet or in some cases provide no correlation. The effects of pectin (from the various genotypes of okra) as disintegrants (∼5%, ∼7.5%, and ∼10% w/w) on the crushing strength of formulated tablets have been illustrated in Figure 3. Pectin from PC1, PC2, and PC5 as disintegrants exhibited similar properties to those of the standard (maize starch BP). There was an increase in the hardness of the tablets prepared from these batches as their concentrations increased. This is similar to studies carried out by [1] on some selected disintegrants. This suggests that for disintegrants exhibiting such properties a careful selection of lower concentrations of disintegrant is needed for the preparation of a tablet with good mechanical characteristics since higher concentrations tend to increase the bonding strength in tablets and can result in very compact tablets. However, PC4 and PC3 showed a reduction in tablet hardness from 7.5% to 10% w/w. This reduction in tablet hardness is also similar to other studies carried out on disintegrants by [2, 37] suggesting that, generally, some disintegrants tend to weaken tablet structure when used in higher concentrations. These variations in hardness were further confirmed statistically by carrying out a two-tailed test on the various pectin concentrations with their corresponding standard concentrations. Significant differences (*p* ≤ 0.05) were observed between some concentrations and the standard while, in some cases, there were no significant differences (*p* > 0.05) (Figure 4).

A tablet weight loss not exceeding 1% of the tablet weight being tested is usually deemed appropriate for pharmaceutical products [22, 26]. Controlling friability requires accurate control of tablet weight and roughness of tablet surface, good granulation method, good compression method, moisture at low levels, and acceptable concentrations of binders and disintegrants [40]. All the formulated batches passed the friability test (Table 4), indicating that incorporation of okra pectin as a disintegrant does not produce soft tablets, which is a challenge of conventional disintegrants.

**Figure 5:**
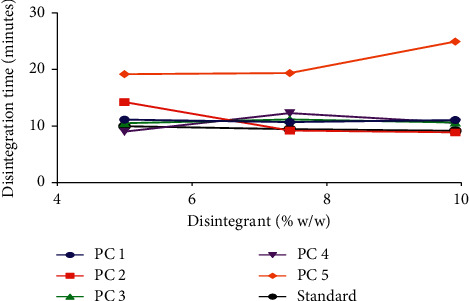
Effect of pectin concentrations as disintegrants on disintegration time.

**Figure 6:**
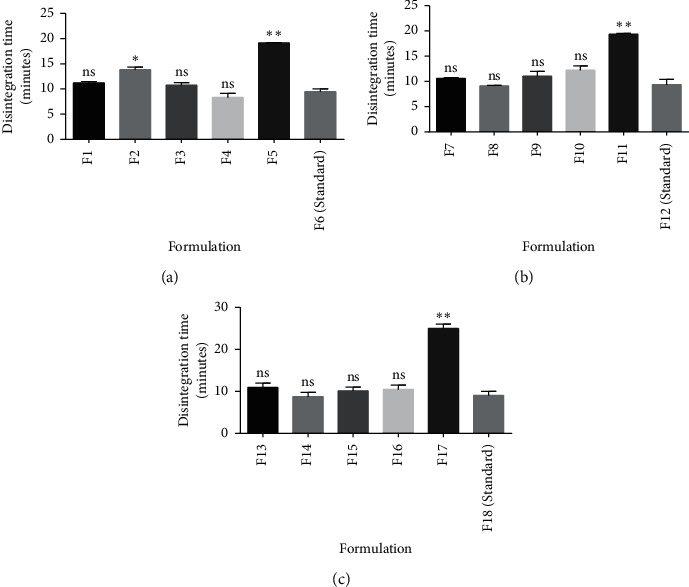
Comparative analysis on the disintegration time of pectin from various genotypes as disintegrants ((a) 5% w/w, (b) 7.5% w/w, and (c) 10% w/w) and a standard disintegrant using *t*-test. Values are mean ± SD (*n* = 2). ^*∗*^*p* ≤ 0.05, ^*∗∗*^*p* ≤ 0.01, and *p* > 0.05 not significant (ns).

Pectin, a natural polysaccharide just as starch, can exhibit potential disintegration property by swelling when it comes into contact with water and this will result in a gradual increase in pressure and stress within the tablet and subsequently break down the tablet. Pectin from PC5 was unable to cause the breakup of uncoated immediate release tablets within the stipulated time range (*D*_*T*_ < 15 min) at all concentrations (5% w/w (F5), 7.5% w/w (F11), and 10% w/w (F17)) used (Table 4, Figure 5). PC5 may have exhibited poor disintegration properties in uncoated immediate release tablets due to slower rate in the uptake of water and formation of a gel plug after imbibition of water [3, 4, 41]. All the other genotypes exhibited good disintegrating properties (*D*_*T*_ ˂ 15 mins) within the concentrations used (Table 4, Figure 5). Generally, an increase in disintegrant concentration may have two effects on disintegration time: a reduction in disintegration time if the concentrations used are below the optimal concentration or no correlation with disintegration time if the concentrations used are equal to or above the optimal concentration [1, 3, 4, 29]. PC1, PC2, and the standard disintegrant (Figure 5) produced a reduction in disintegration time with an increase in concentration. However, the disintegration times of PC4 and PC3 did not decrease with an increase in concentration indicating that their optimal disintegration times were obtained at 5% w/w (Figure 5). These observations were confirmed by variations in statistical analysis between the disintegration times of the standard disintegrant and the okra pectins (Figure 6).

**Figure 7:**
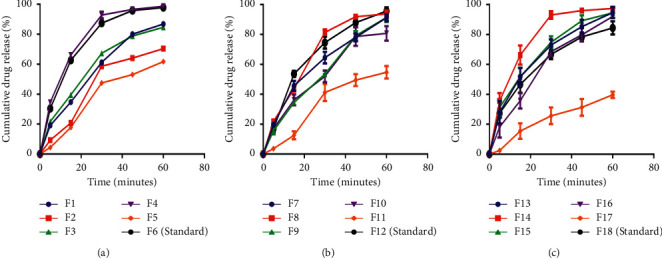
Dissolution profiles of formulated paracetamol tablets containing (a) ∼5% w/w okra pectin, (b) ∼7.5% w/w okra pectin, and (c) ∼10% w/w okra pectin in phosphate buffer pH 5.8 (mean ± SD, *n* = 3).

The dissolution profiles of the paracetamol tablets containing different concentrations of the okra pectin in phosphate buffer pH 5.8 are shown in Figure 7. It was observed that, within 45 minutes of dissolution, all the tablet batches passed the dissolution test (˃70% released) as specified by the British Pharmacopoeia with the exception of F2, F5, F11, and F17. Disintegration precedes dissolution hence disintegrants which are able to cause a faster breakup of immediate release tablets will enhance the dissolution process of the active ingredient and produce tablets with good drug release profiles. PC5 at all concentrations as disintegrants in batches F5, F11, and F17 failed the disintegration test and subsequently the dissolution test and hence will not be suitable as a disintegrant for immediate release tablets. However, the delay in disintegration action of pectin from PC5 may suggest its potential as a good control releasing agent at low concentrations. Pectin from PC2 when used as a disintegrant (5 % w/w) in batch F2 produced a disintegration time very close to the limit of 15 minutes and subsequently resulted in a poor dissolution profile which did not correspond to the expected drug release. An increase in the concentration of PC2 subsequently reduced disintegration time and produced tablets with good release profiles which complied with the standard limit [26] (˃70% drug release in 45 minutes).

Formulations which passed the dissolution profile were then fitted into the difference and similarity factors equations [42–44] and their results are shown in Table 5. From the results obtained, the batches of formulations F4, F7, F8, F13, F15, and F16 had similar dissolution profiles as formulations with maize starch BP incorporated as disintegrant. Thus, at a disintegrant concentration of 5% w/w, okra pectin from PC4 was able to release paracetamol from the tablet to a similar extent as maize starch BP. At a disintegrant concentration of 7.5% w/w, okra pectins from PC1 and PC2 were able to have similar effects on the release of paracetamol from the formulation as maize starch BP. At a disintegrant concentration of 10% w/w, okra pectins from PC1, PC3, and PC4 achieved similar paracetamol release as maize starch BP. The various okra pectins in their respective concentrations can be considered as alternate disintegrants to maize starch BP in the formulation of bioequivalent immediate release tablets [45]. Since these genotypes of okra are readily available in Ghana, their pectins can be harnessed and used to formulate bioequivalent products at a lower cost in comparison to the imported maize starch BP.

## 4. Conclusion

Variations in genotypes of okra have an influence on pectin yield. The extracted okra pectins contained negligible amounts of toxic metals and appreciable levels of micronutrients; hence, they can be used as potential pharmaceutical excipients. Pectins extracted from PC4, PC1, and PC3 when used as disintegrants in the range of 5–10% w/w exhibited good disintegrating properties in immediate release tablets and are comparable to maize starch BP. Pectin from PC2 produces good disintegrating properties in immediate release tablets within concentration ranges of 7.5–10% w/w. Pectin extracted from PC5 does not possess the desired disintegrating properties in immediate release tablets within the concentrations used (5–10% w/w). Pectin obtained from different genotypes of okra has an influence on its disintegrating and tablet formulation characteristics.

## Figures and Tables

**Table 1:** Composition of formulated batches.

Formulation code	Ingredients (mg/tablet)				
	Disintegrant	Disintegrant	Binder	Diluent	Lubricant and glidant
	Pectin	Starch	Tragacanth	Lactose	Talc
F1–F5	31.0	—	7.5	89.0	2.5
F6	—	31.0	7.5	89.0	2.5
F7–F11	46.5	-	7.5	73.5	2.5
F12	—	46.5	7.5	73.5	2.5
F13–F17	62.0	—	6.5	58.0	3.5
F18	—	62.0	6.5	58.0	3.5

Paracetamol (500 mg) was used as the active pharmaceutical ingredient (API) in each formulation. Batches F1–F5 contained pectin from the various genotypes in the order PC1–PC5. Batches F7–F11 contained pectin from the various genotypes in the order PC1–PC5. Batches F13–F17 contained pectin from the various genotypes in the order PC1–PC5.

**Table 2:** Physicochemical properties of the extracted pectin.

Parameter	Okra genotypes				
	PC1	PC2	PC3	PC4	PC5
Pectin yield (%)	6.12	18.84	11.93	7.7	13.54
pH	6.92 ± 0.010	6.74 ± 0.006	6.39 ± 0.012	6.87 ± 0.006	6.80 ± 0.010
Moisture content (%)	16.2 ± 1.20	19.7 ± 0.5	11.2 ± 2.0	16.1 ± 0.6	15.9 ± 0.00
Swelling index (%)	405.05 ± 0.045	555.737 ± 0.015	407.246 ± 0.009	441.666 ± 0.020	540.394 ± 0.010
Solubility					
Hot water	Sparingly soluble	Sparingly soluble	Sparingly soluble	Sparingly soluble	Sparingly soluble
Cold water	Sparingly soluble	Sparingly soluble	Sparingly soluble	Sparingly soluble	Sparingly soluble
Pet. ether	Practically insoluble	Very slightly soluble	Very slightly soluble	Practically insoluble	Very slightly soluble
Diethyl ether	Practically insoluble	Sparingly soluble	Slightly soluble	Very slightly soluble	Very slightly soluble
Methanol	Slightly soluble	Practically insoluble	Very slightly soluble	Slightly soluble	Slightly soluble
Ethanol	Very slightly soluble	Very slightly soluble	Very slightly soluble	Slightly soluble	Slightly soluble

**Table 3:** Flow properties of formulated granules.

Formulation code	Hausner ratio	Carr's index (%)	Angle of repose (Ɵ)
F1	1.08	7.71	29.47
F2	1.15	13.66	31.55
F3	1.08	7.99	29.29
F4	1.04	2.21	30.38
F5	1.11	9.89	30.77
F6	1.10	15.87	29.89
F7	1.14	13.95	33.02
F8	1.18	15.40	28.78
F9	1.14	12.24	28.61
F10	1.09	8.51	28.61
F11	1.15	15.33	28.95
F12	1.18	15.48	30.08
F13	1.06	5.99	30.00
F14	1.09	7.30	30.00
F15	1.07	6.80	30.00
F16	1.07	6.80	30.00
F17	1.11	7.80	30.00
F18	1.01	10.56	29.00

**Table 4:** Physical and mechanical properties of paracetamol batches produced using different concentrations of the okra pectin as disintegrant.

Formulation code	Average weight (g) *n* = 20	Tablet thickness (mm) *n* = 20	Tablet diameter (mm) *n* = 20	Hardness (kg/F) *n* = 6	Friability (%)	Disintegration time (*D*_*T*_) (min) *n* = 2	Drug content (%) *n* = 3
F1	0.630 ± 0.003	3.70 ± 0.093	13.07 ± 0.235	5.60 ± 0.539	0.330	11.40 ± 0.265	99.61 ± 0.03
F2	0.630 ± 0.001	3.98 ± 0.073	13.04 ± 0.009	4.88 ± 1.308	0.139	14.20 ± 0.398	99.61 ± 0.07
F3	0.620 ± 0.001	3.78 ± 0.145	13.03 ± 0.015	7.38 ± 2.46	0.005	10.40 ± 0.447	100.12 ± 0.01
F4	0.620 ± 0.001	3.61 ± 0.092	13.06 ± 0.025	5.92 ± 1.264	0.002	8.90 ± 0.566	99.92 ± 0.10
F5	0.621 ± 0.002	3.96 ± 0.104	13.08 ± 0.051	6.44 ± 1.405	0.011	19.20 ± 0.100	99.82 ± 0.03
F6	0.620 ± 0.001	3.76 ± 0.099	13.07 ± 0.025	4.02 ± 0.861	0.005	9.87 ± 0.640	99.75 ± 0.08
F7	0.630 ± 0.002	3.74 ± 0.043	13.03 ± 0.011	6.4 ± 0.57	0.000	10.60 ± 0.148	99.82 ± 0.10
F8	0.632 ± 0.002	3.87 ± 0.132	13.02 ± 0.085	4.92 ± 0.719	0.002	9.09 ± 0.122	99.30 ± 0.04
F9	0.629 ± 0.002	3.88 ± 0.112	13.04 ± 0.015	8.14 ± 2.519	0.002	11.08 ± 0.898	97.46 ± 0.01
F10	0.631 ± 0.002	3.95 ± 0.109	13.05 ± 0.017	8.60 ± 2.742	0.005	12.22 ± 0.867	98.26 ± 0.03
F11	0.631 ± 0.005	3.94 ± 0.134	13.04 ± 0.017	6.74 ± 2.703	0.009	19.39 ± 0.145	99.59 ± 0.11
F12	0.621 ± 0.002	3.78 ± 0.087	13.05 ± 0.401	4.62 ± 0.563	0.174	9.39 ± 1.07	99.56 ± 0.03
F13	0.629 ± 0.001	3.68 ± 0.094	13.05 ± 0.012	6.56 ± 0.467	0.265	10.98 ± 0.989	99.71 ± 0.01
F14	0.621 ± 0.002	3.87 ± 0.068	13.09 ± 0.025	6.20 ± 0.663	0.742	8.78 ± 1.023	99.00 ± 0.10
F15	0.630 ± 0.003	3.67 ± 0.096	13.07 ± 0.018	7.34 ± 2.062	0.017	10.53 ± 0.895	97.63 ± 0.20
F16	0.630 ± 0.002	3.72 ± 0.203	13.04 ± 0.008	3.74 ± 1.152	0.002	10.49 ± 1.023	99.29 ± 0.03
F17	0.631 ± 0.003	3.88 ± 0.138	13.08 ± 0.013	7.10 ± 0.866	0.256	25.04 ± 0.987	101.36 ± 0.02
F18	0.639 ± 0.002	3.82 ± 0.067	13.08 ± 0.045	7.42 ± 0.287	0.196	9.05 ± 0.989	99.00 ± 0.11

**Table 5:** Difference (*f*1) and similarity (*f*2) factors of formulated batches.

Formulation	Difference factor (*f*1)	Similarity factor (*f*2)	Comment
F1	25.23	34.62	Dissimilar
F3	21.86	38.18	Dissimilar
F4	3.28	72.38	Similar
F7	9.06	53.68	Similar
F8	6.77	65.59	Similar
F9	24.29	40.20	Dissimilar
F10	20.74	39.94	Dissimilar
F13	9.17	59.10	Similar
F14	29.23	35.94	Dissimilar
F15	12.6	53.8	Similar
F16	10.21	56.44	Similar

## Data Availability

The data used to support the findings of this study are included in the article and also available from the corresponding author upon request.
